# Magnetic Second‐Order Topological Insulators in 2H‐Transition Metal Dichalcogenides

**DOI:** 10.1002/advs.202301952

**Published:** 2023-07-30

**Authors:** Guodong Liu, Haoqian Jiang, Zhenzhou Guo, Xiaoming Zhang, Lei Jin, Cong Liu, Ying Liu

**Affiliations:** ^1^ State Key Laboratory of Reliability and Intelligence of Electrical Equipment Hebei University of Technology Tianjin 300130 China; ^2^ School of Materials Science and Engineering Hebei University of Technology Tianjin 300130 China

**Keywords:** 2D material, ferromagnetic, higher order topological insulator

## Abstract

The transition metal dichalcogenides, 2H‐VX_2_ (X = S, Se, Te), are identified as two‐dimensional second‐order topological insulator (SOTI) with a ferromagnetic ground state by first‐principles calculations. The 2H‐VX_2_ (X = S, Se, Te) materials have a nontrivial band gap in two spin channels is found and exhibit topologically protected corner states with spin‐polarization. These corner states only accommodate the quantized fractional charge (e/3). And the charge is bound at the corners of the nanodisk geometry 2H‐VX_2_ (X = S, Se, Te) in real space. The corner states are robust against symmetry‐breaking perturbations, which makes them more easily detectable in experiments. Further, it is demonstrated that the SOTI properties of 2H‐VX_2_ (X = S, Se, Te) materials can be maintained in the presence of spin‐orbit coupling and are stable against magnetization. Overall, the results reveal 2H‐VX_2_ (X = S, Se, Te) as an ideal platform for the exploration of magnetic SOTI and suggest its great potential in experimental detection.

## Graphical abstract

2H‐VX_2_ (X = S, Se, Te) are identified as a two‐dimensional second‐order topological insulator with a ferromagnetic ground state by first‐principles calculation. They also show a topologically protected corner state with spin‐polarization. Remarkably, these corner states are robust against symmetry‐breaking perturbations, which makes them more easily detectable in experiments.

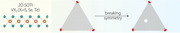

## 1 Introduction

Topological insulator (TI) has been one of the most active fields in condensed matter physics since their discovery.^[^ ^1^, ^2^, ^3^, ^4^, ^5^, ^6^, ^7^, ^8^ ^]^ Conventionally, the most unusual feature of a *d‐*dimensional TI is the appearance of protected gapless states on its (*d*‐1)‐dimensional boundary. Recently, the concept of second‐order topological insulator (SOTI) has attracted great interest. SOTI is featured by the gapless states on its (*d*‐2)‐dimensional boundary. For example, three‐dimensional (3D) SOTI has one‐dimensional (1D) hinge states in gapped surface states; And two‐dimensional (2D) SOTI has zero‐dimensional (0D) corner states in gapped edge states. In order to accurately evaluate whether a material is SOTI, some new bulk topological invariants with corresponding symmetries are used.^[^ ^9^, ^10^, ^11^, ^12^, ^13^, ^14^, ^15^, ^16^ ^]^ So far, SOTIs have been predicted in some nonmagnetic^[^ ^17^, ^18^, ^19^, ^20^ ^]^ and magnetic^[^ ^21^, ^22^, ^23^ ^]^ 3D materials, while 2D SOTIs have been found mainly in nonmagnetic materials.^[^ ^24^, ^25^, ^26^ ^]^

In 2D materials, transition‐metal dichalcogenides (TMDs) are a class of materials that have garnered significant attention due to their exceptional performance in electronics, electrocatalysts, optical properties, and rich valley‐related physics.^[^ ^27^, ^28^, ^29^, ^30^, ^31^, ^32^ ^]^ In addition to these properties, 2D TMDs have also been predicted to host a novel topological electronic structure. Recently, 0D corner states were discovered in the band gap of hexagonal TMDs (h‐TMDs) with a triangular prism structure. The robust (*d*‐2)‐dimensional boundary correspondence in h‐TMDs is protected by the rotation symmetry.^[^ ^33^ ^]^ Interestingly, based on the hidden breathing Kagome lattice (BKL) structure mimicked by the *d*‐orbitals from the transition metal atoms, this nontrivial topology in h‐TMDs can be explained by the higher‐order topological insulators concept in BKL.^[^ ^34^ ^]^ In Ref,^[^ ^33^ ^]^ it was also revealed that the band inversion of the *d* orbitals indicates a nontrivial Wannier center (WC), which is a signature of SOTI for the 2D h‐TMDs. And they used these theoretical analyses to confirm that some h‐TMDs materials, such as 2D MX_2_ (M = Mo, W; X = S, Se, Te), are indeed SOTIs. In comparison to graphdiyne and 2D hexagonal group‐V materials, 2D TMDs have a larger band gap (≈1.7 eV), facilitating separation of corner states from edge/bulk states in their electronic band structure. Notably, transition metal ions that have partially filled *d* shells are commonly associated with magnetism. Given the advantages of TMDs in nonmagnetic SOTI systems, magnetic TMDs may provide an ideal platform to study magnetic SOTI.

Recent experimental and theoretical studies have suggested the presence of ferromagnetism in single‐layer TMDs.^[^ ^35^, ^36^, ^37^, ^38^ ^]^ In particular, vanadium‐based dichalcogenides such as VX_2_ (X = S, Se, and Te) have been found to exhibit inherent ferromagnetism at room temperature and semiconducting properties in their 2H‐phase monolayer structure. In addition to the above characteristics, 2H‐VX_2_ (X = S, Se, and Te) also has a triangular prism structure, so 2H‐VX_2_ (X = S, Se, and Te) is expected to be a favorable candidate for magnetic SOTI. Moreover, the monolayer 2H‐VS_2_ has been successfully synthesized in the experiment,^[^ ^39^ ^]^ which enables the experimental detection of properties in the magnetic SOTI soon.

In this work, we show that VX_2_ (X = S, Se, and Te) exhibit a ferromagnetic ground state and are magnetic 2D SOTIs. The nontrivial topology and 0D corner states in these materials are protected by the rotation symmetry of the lattice. Based on the first‐principles calculations, we confirm that VX_2_ (X = S, Se, and Te) have gapped bulk and edge states in two spin channels. Notably, the spin‐polarized corner states can be extracted in the gap. By symmetry analysis, we verify that the corner states carry a quantized fractional charge protected by *C*_3_ rotation symmetry. We also explore the stability of corner states and find that the SOTI phase is robust to symmetry‐breaking perturbations. The SOTI properties in VX_2_ (X = S, Se, and Te) can still be preserved even if holes are introduced to break *C*_3_ symmetry or spin‐orbit coupling (SOC) is included to break T symmetry. And the corner states accommodating a quantized fractional charge remain to be localized at the corner of the sample.

Since the electronic band structures for all the VX_2_ (X = S, Se, and Te) have a similar topology as well as the monolayer 2H‐VS_2_ has already been synthesized in experiment. So, as a typical example, we will focus on the results obtained for monolayer 2H‐VS_2_. The results for other materials are presented in Supporting Information.

## 2 Computational Details

This work is based on density functional theory (DFT)^[^ ^40^ ^]^ for first principles calculations by Vienna Ab initio Simulation Package (VASP).^[^ ^41^ ^]^ The exchange‐correlation potential is treated with the generalized gradient approximation (GGA)^[^ ^42^ ^]^ of Perdew‐Burke‐Ernzerhof function.^[^ ^43^ ^]^ Projector augmented wave potentials are provided by VASP and have been used on all elements. For the monolayer 2H‐VS_2_ crystal structure, a large vacuum space of 20 Å is established to avoid potential interactions between the layers. In our calculations, the cutoff energy is set as 500 eV. The Brillouin zone is sampled by a Monkhorst‐Pack k mesh with a size of 9 × 9 × 1. To optimize the crystal structure, the force and energy convergence criteria are set as 0.001 eV Å^−1^ and 10^−6^ eV, respectively. To investigate the dynamical stability of the monolayer 2H‐VS_2_, the phonon spectrum is calculated by the PHONOPY code.^[^ ^44^ ^]^ Since V is a transition metal element, the Coulomb interaction (U) between electrons is considered and GGA+U is used to calculate the band structure.^[^ ^45^ ^]^ In the main text, the calculation results are based on the effective Coulomb energy, U_eff_, set as 2.5 eV. The conclusions in this work do not change over a large range of U values (0–4 eV) (See Figure S1, Supporting Information). The edge states are calculated by the Wannier Tools package.^[^ ^46^ ^]^ The tight‐binding model of the triangular nanodisk is constructed by the Pybinding software package,^[^ ^47^ ^]^ so as to calculate the discrete energy spectra of the nanodisk and further observe the local charge states of the corner states in the gap.

## 3 Results and Discussion

### 3.1 Structure, Stability and Magnetism

**Figure 1:**
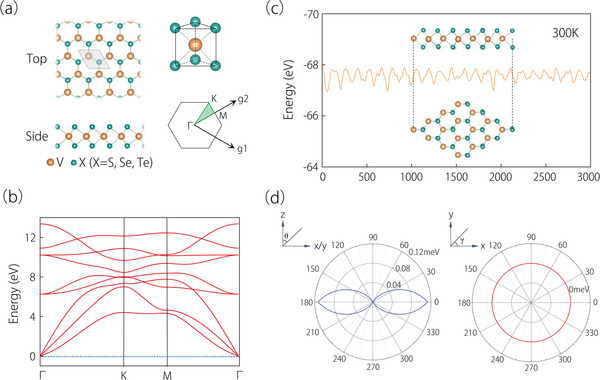
a) Top and side views of the monolayer 2H‐VS_2_, alongside its triangular primitive cell. The first Brillouin zone with high‐symmetry points is given. b) A phonon dispersion of the monolayer 2H‐VS_2_. A 2 × 2 × 1 supercell is used in the calculation. c) The monolayer 2H‐VS_2_ during AIMD simulations at 300 K; the inset shows the final state of the structure. d) The MAE upon rotating the spin within the *z–x/y* and *x–y* planes, where θ and γ are the polar and azimuthal angles, respectively.

The lattice structure of 2H‐VX_2_ (X = S, Se, and Te) is depicted in **Figure** **1**a. The lattice structure is constructed by stacked X‐V‐X atom layers. The basal plane is comprised of V atoms and two layers of X atoms are situated on both sides of the basal plane. Each V atom is boned to six X atoms. The lattice structure belongs to space group $P\bar{6}m2$, and the corresponding point group is *D*_3*h*_ generated by *C*_3_ and *C*_2_′. The primitive cell contains one V and two X atoms, and the lattice constant for the monolayer 2H‐VX_2_ (X = S, Se, and Te) is provided in Table S2 (Supporting Information). The lattice parameters of monolayer 2H‐VS_2_ are consistent with the previous report.^[^ ^48^ ^]^

The monolayer 2H‐VS_2_’s stability can be assessed through its phonon spectrum and thermal stability. Figure 1b shows that there are no imaginary frequencies present in the entire Brillouin zone, which indicates the dynamical stability of monolayer 2H‐VS_2_. In addition, spin‐polarized *ab* initio molecular dynamics (AIMD) simulations are carried out in a 2 × 2 × 1 supercell to evaluate the thermal stability of the monolayer 2H‐VS_2_. Figure 1c shows that there are no bond breakages or geometric reconfigurations in the final states after 3000 steps at 300 K, which suggests that monolayer 2H‐VS_2_ is thermally stable. In addition, we have also investigated the stability of monolayer 2H‐VSe_2_ and 2H‐VTe_2_ (see Figure S6, Supporting Information). Our results show monolayer 2H‐VSe_2_ and 2H‐VTe_2_ also have excellent dynamical and thermodynamic stabilities.

It is worth noticing that the monolayer 2H‐VS_2_ always preserves in a ferromagnetic ground state regardless of the U value (as demonstrated in Figure S2, Supporting Information). The magnetization of monolayer 2H‐VS_2_ is mainly contributed by V atoms (Figure S3, Supporting Information). The obtained magnetic moment of V atom is ≈1.3 μ_*B*_ with U = 2.5 eV (see Table S1, Supporting Information). Previous work has predicted that the Curie temperature (*T_c_*) is ≈ 292 *K* for the monolayer 2H‐VS_2_ (see Table S2, Supporting Information for the other 2H‐VX_2_ (X = S, Se, and Te) materials).^[^ ^37^ ^]^ Before studying its electronic band structure, we also determine an easy plane of its ferromagnetic state by using the DFT+U+SOC calculations. Figure 1d shows the magnetocrystalline anisotropy energy (MAE) achieved by rotating the magnetization direction in the *zox* (*zoy*) and *xoy* planes. These results clearly indicate that the in‐plane (xy‐plane) magnetization dominates in monolayer 2H‐VS_2_.

### 3.2 Electronic Band Structure

**Figure 2:**
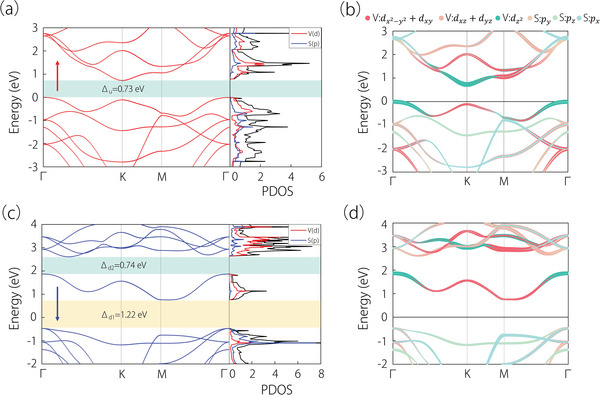
a,c) Electronic band structures for the spin‐up and spin‐down states in the absence of SOC, alongside the PDOS. b,d) Electronic band structures with projections onto atomic orbitals for spin‐up and spin‐down states, respectively.

The electronic band structure and partial density of states (PDOS) of different spin channels for the monolayer 2H‐VS_2_ are given in **Figure** **2**a,c. It is observed that both spin channels exhibit semiconductive band structure with indirect band gaps of approximately 0.73 and 1.22 eV for spin‐up (Δ_u_) and spin‐down (Δ_d1_) channels, respectively. Akin to monolayer MX_2_ (M = Mo, W; X = S, Se, Te),^[^ ^49^ ^]^ Bloch states near the band edges for the conduction and valence bands in the spin‐up channel are primarily contributed by the *d*‐orbitals of the transition metal atom (in this case, V). This is demonstrated in Figure 2b. In addition, one can observe that there is a band inversion between ($d_{x^{2}-y^{2}},\;d_{xy}$) and $d_{z^{2}}$ for spin‐up states at K point. Following the work of Fang et al^[^ ^50^ ^]^ and Jung et al,^[^ ^33^ ^]^ if the model has *C*_3_, its polarization can be directly deduced from the eigenvalues of *C*_3_, giving rise to a nontrivial WC position, as shown in Figure S4 (Supporting Information). By analogy, the band gap (Δ_d2_ ≈ 0.74 eV) in the spin‐down channel is also nontrivial due to the presence of the *d*‐orbitals inversion.

In addition, the nontrivial topology for the band gaps Δ_u_ and Δ_d1_ can be attributed to the symmetry protection. It is necessary to point out that the spin‐up and spin‐down states are decoupled, which is a natural consequence of the absence of SOC in a FM state, such that the system preserves all the original symmetries in the absence of FM, including the time‐reversal symmetry. Consequently, the presence of *C*_3_ rotation symmetry allows for the existence of corner states with quantized fractional charge, which can be derived from the *C*_3_ symmetry eigenvalues of the occupied bands at high‐symmetry points (HSPs) in the Brillouin zone.^[^ ^51^ ^]^ We denote HSPs as Π^(3)^, and the superscript refers to the threefold rotation symmetry. Thus, the eigenvalues of *C*_3_‐symmetry at HSPs can be expressed as

(1) $$\Pi_{p}^{\left(3\right)}=e^{\frac{2\pi i\left(p-1\right)}{3}},\;p\;=\;1,\;2,\;3$$

As a result, the topological index $\left[\Pi_{p}^{\left(3\right)}\right]$ can be defined based on the eigenvalues of *C*_3_ compared to a reference point **Γ**  =  (0, 0), explicitly given by

(2) $$\left[\Pi_{p}^{\left(3\right)}\right]=\#\Pi_{p}^{\left(3\right)}-\#\Gamma_{p}^{\left(3\right)}$$

Here, $\#\Pi_{p}^{\left(3\right)}$ $\left(\#\Gamma_{p}^{\left(3\right)}\right)$ is the number of occupied bands with corresponding $\Pi_{p}^{\left(3\right)}$ $\left(\Gamma_{p}^{\left(3\right)}\right)$. The topological indexes for both spin channels at HSPs have been listed in **Table** **1**. The quantized fractional corner charge $\left(Q_{corner}^{\left(3\right)}\right)$ can be calculated using the following formula:

(3) $$Q_{corner}^{\left(3\right)}=\frac{\left|e\right|}{3}{(n}_{1a}^{\mathrm{ion}}-\nu )-\frac{\left|e\right|}{3}{[K}_{1}^{\left(3\right)}],\;\mathrm{mod}\;e$$

Here, $n_{1a}^{ion}$ stands for the ionic number at Wykoff position *1a*, and ν is the number of occupied bands, *e* is the charge of a free electron. For the monolayer 2H‐VS_2_, we derive the topological index $\left[K_{p}^{\left(3\right)}\right]=\left(-2,0\right)\left(p\;=\;1,\;2\right)$, and $Q_{corner}^{\left(3\right)}=e/3$ for both spin‐up and spin‐down states. The corner charges are also calculated for the other 2H‐VX_2_ (X = S, Se, and Te) materials, which are shown in Table S2 (Supporting Information).

**Table 1:** Topological invariants and a corner charge of the monolayer 2H‐VS_2_

State	$n_{1a}^{\left(\mathrm{ion}\right)}$	ν	${[K}_{1}^{\left(3\right)}]$	${[K}_{2}^{\left(3\right)}]$	${[K}_{1}^{{}^{\prime }\left(3\right)}]$	${[K}_{2}^{{}^{\prime }\left(3\right)}]$	$Q_{Corner}^{\left(3\right)}$
Spin‐up	9	9	−2	0	–	–	$\frac{e}{3}$
Spin‐down	8	8	−2	0	–	–	$\frac{e}{3}$
SOC	17	17	−3	2	−2	−2	$\frac{e}{3}$

### 3.3 Corner States

**Figure 3:**
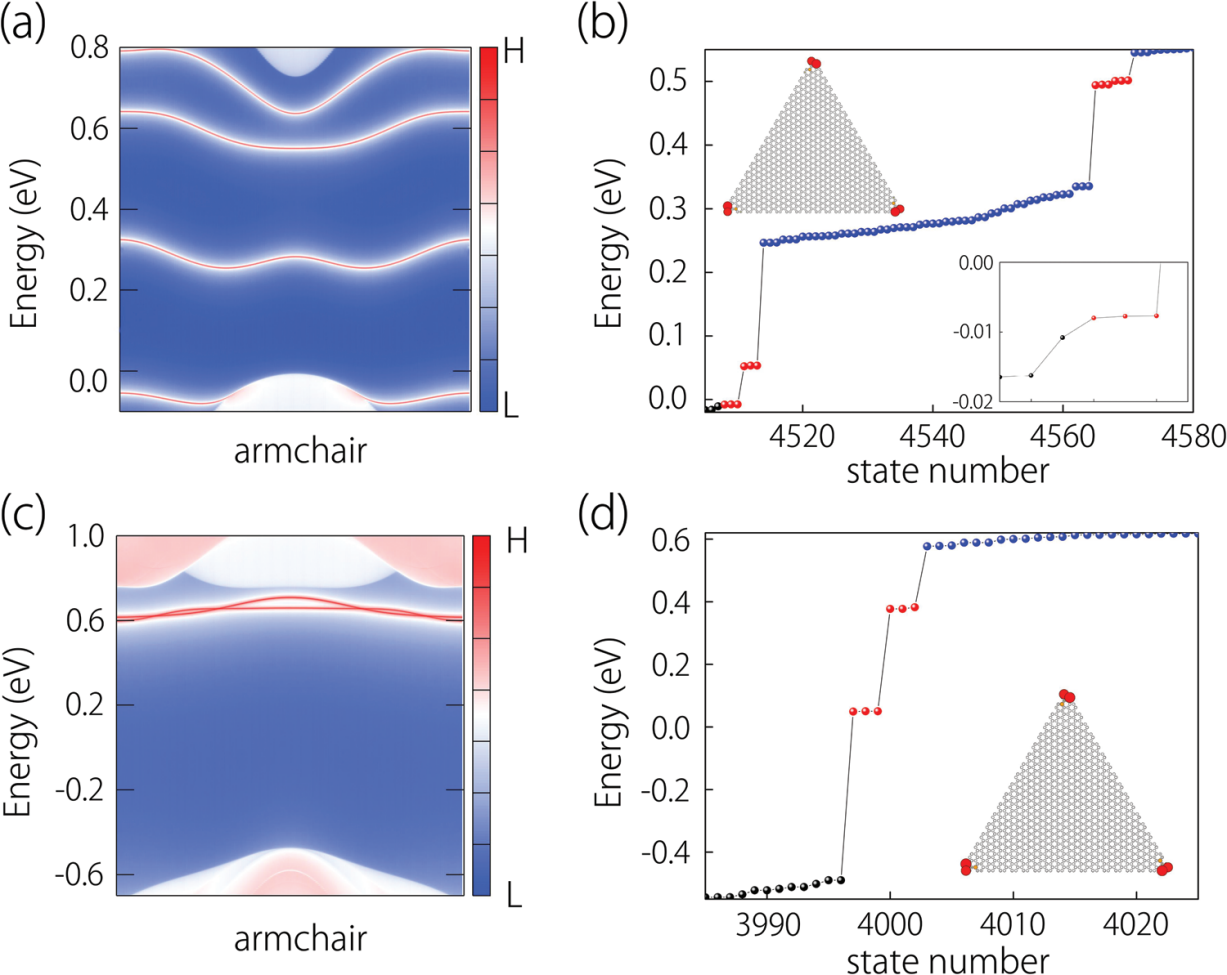
a,c) Surface states for spin‐up and spin‐down states on the armchair boundary, respectively. b,d) Spectra for the triangle nanodisk geometry with an armchair edge for spin‐up and spin‐down states. The insets show the distribution of the corner states in the spectra (marked by red dots). An enlarged view of the corners states around the Fermi level is given in (b). The black, blue, and red dots stand for the respective bulk, edge, and corner states.

To demonstrate the presence of topological corner states, we construct the tight‐binding (TB) model for the monolayer 2H‐VS_2_ in the form of a triangle nanodisk that preserves the *C*_3_‐symmetry. The electronic band structure obtained from TB calculations is consistent with the result derived from DFT calculations, as shown in Figure S8 (Supporting Information), which indicates that the constructed TB model is completely reliable. It is necessary to consider the different boundaries when nanodisks are constructed because the topology in symmetry protection is sensitive to the boundary geometry. By calculating the edge states of the triangle nanodisks with the zigzag and armchair boundaries, we find that monolayer 2H‐VS_2_ has a zigzag metallic edge (as seen in Figure S9, Supporting Information) similar to MoS_2_,^[^ ^52^, ^53^ ^]^ whereas the states along armchair boundary exhibit gapped edge states (see in **Figure** **3**a,c). The presence of edge states inside the bulk gap indicates that monolayer 2H‐VS_2_ is not a conventional insulator, and the gap in the edge states also hints at its difference from the conventional TI. Indeed, we discovered two pairs of three degenerate corner states in the edge gaps for the spin‐up and spin‐down states, as shown in Figure 3b,d, respectively. The Corner states for two spin channels appear at different energy levels and exhibits a spin‐polarized feature. We also calculate the corner states appearing in Δ_d2_ for the spin‐down channel, as shown in Figure S10 (Supporting Information). By checking their positions in real space, we found that these corner states are localized at the corners of the triangle nanodisk, as shown in the illustrations in Figure 3b,d. In addition, by comparing the corner state distribution of nanodisks with different sizes, we confirm that the size of the nanodisk used in this work has excluded the finite size effect between the corners (See Figure S11, Supporting Information). The nontrivial topological indexes together with corner states with quantized fractional charge demonstrate that the monolayer 2H‐VS_2_ is a magnetic 2D SOTI.

**Figure 4:**
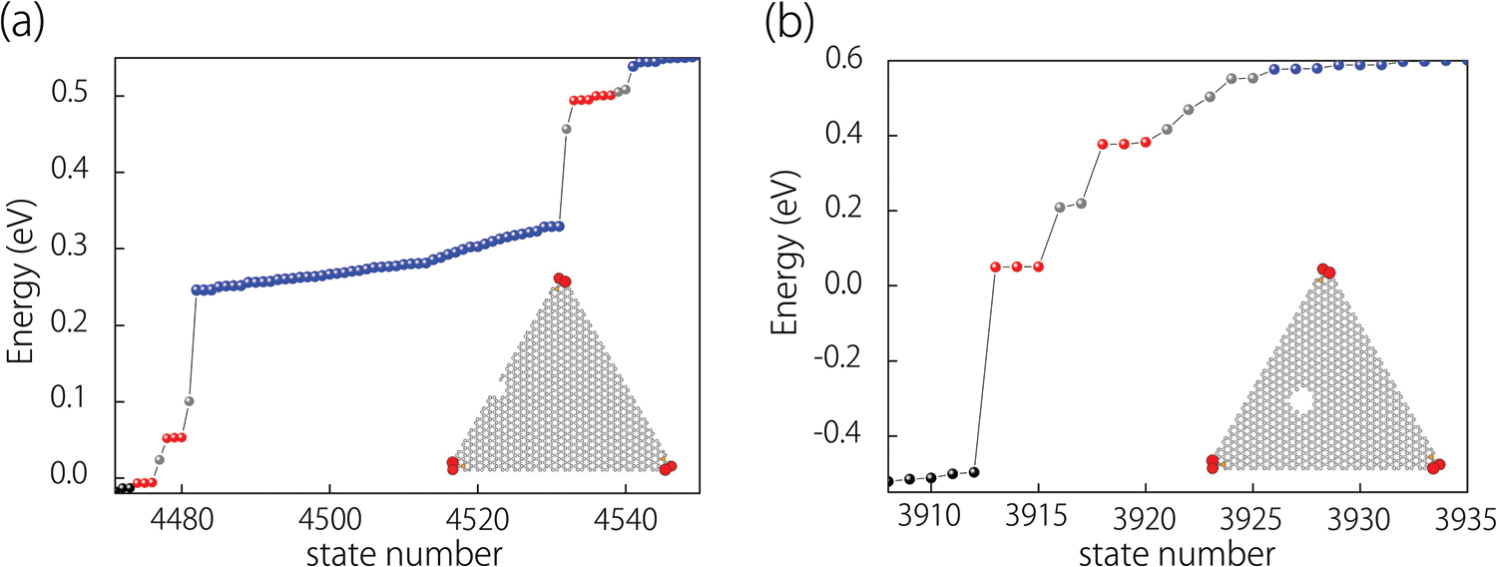
a,b) shows corresponding calculated energy spectra for spin‐up and spin‐down states with artificial distortions that break the threefold rotation symmetry in the monolayer 2H‐VS_2_. The insets also present the corner states (marked by red dots) located at the corners. The gray dots refer to the emergent 0D states at distortions.

Next, we demonstrate the robustness of the corner states to perturbations that break the crystal symmetry. To achieve this, an artificial hole was introduced at the edge or internal region of the triangle nanodisk to break the *C*_3_‐symmetry, and then the corner states were recalculated. It was found that the corner states with the same energy and degeneracy still exist in the gap well preserved, as shown in **Figure** **4**a,b. This is mainly because the symmetry‐breaking perturbations are far away from the corners of the nanodisk and a generalized *C*_3_ symmetry still dominates. Upon checking the spatial positions of the corner states, we confirm that they are still localized at the original position, namely, the corners of the nanodisk. The robustness of these corner states would greatly facilitate their experimental detection in the future. One may notice that some additional 0D states are also appearing in the gap (marked by green dots in Figure 4a,b). As shown in Figure S12 (Supporting Information), these 0D states are pinned at the boundary exposed by the artificial hole rather than the topological corner states.

**Figure 5:**
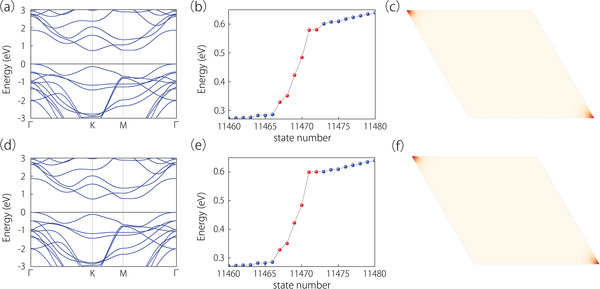
a,d) Electronic band structures for the monolayer 2H‐VS_2_ containing SOC with out‐of‐plane and in‐plane magnetization, respectively. b,e) Show the corresponding energy spectra based on parallelogram nanodisks. The blue and red dots mark the respective edge and corner states. c,f) The distribution of corner states in real space when the directions of magnetization are out‐of‐plane and in‐plane.

Furthermore, we would like to explore the effects of breaking other symmetries in monolayer 2H‐VS_2_. As monolayer 2H‐VS_2_ has magnetism with an energy difference of 110 µeV between in‐plane and out‐of‐plane magnetization, we can test its robustness by introducing SOC with different magnetization directions. When the magnetization direction is out‐of‐plane, *T*‐symmetry is broken while *C*_3_ symmetry is preserved. The corresponding band structure is shown in **Figure** **5**a. Like the case in the absence of SOC, we applied a formula to calculate the corner charge with SOC, as given by,^[^ ^54^ ^]^

(4) $$\begin{array}{ccc} Q_{corner}^{\left(3\right)} & = & \frac{\left|e\right|}{3}\left(n_{1a}^{\mathrm{ion}}-\nu -\left[K_{1}^{\left(3\right)}\right]-\left[K_{2}^{\left(3\right)}\right]-\left[K_{1}^{{}^{\prime }\left(3\right)}\right]-\left[K_{2}^{{}^{\prime }\left(3\right)}\right]\right)\\ & & \times \;\left(\mathrm{mod}\;e\right) \end{array}$$

For the monolayer 2H‐VS_2_, $Q_{corner}^{\left(3\right)}=e/3$ is achieved as well, indicating a nontrivial topology. To check the corner states, a 14 × 14 supercell was constructed by Wannier Tools to form a parallelogram nanodisk. The wave function distribution is plotted in Figure 5b. There are corner states observed within the band gaps, which indicates the SOTI nature in the presence of SOC. It should be noted that the constructed parallelogram nanodisk does not possess the *C*_3_ symmetry. The degeneracy associated with *C*_3_ symmetry was destroyed. Consequently, corner states appear randomly within the band gap. However, in real space, the corner states are still localized at the corners of the nanodisk, as shown in Figure 5c. When the magnetization direction is changed from out‐of‐plane to in‐plane, both the *C*_3_ and T symmetries are broken. However, the band gap remains open during the rotation of the magnetization direction, as depicted in Figure 5d and Figure S13 (Supporting Information). It is well‐known that the topological classification remains unchanged as long as the band gap does not close. The wave function distribution presented in Figure 5e reveals the presence of nondegenerate corner states within the band gaps. These corner states still appear at the corners of the nanodisk, as demonstrated in Figure 5f. Therefore, it can be concluded that, for a SOTI, if the bulk and edge gap is not closed, the corner state can still exist even though the corresponding symmetry is broken by the disturbance. So, corner states in 2D SOTIs are highly robust. The existence cases of corner states (CS) of SOTI are gathered in **Table** **2** when different symmetries are broken.

**Table 2:** The existence of corner states (CS) of SOTI when different symmetries are broken

symmetry	Without SOC	Hole defect	SOC+M_[001]_	SOC+M_[100]_
*C*_3_	✓	×	✓	×
T	✓	✓	×	×
CS	✓	✓	✓	✓

### 3.4 Additional Discussions

To be noted, the SOTI states have already been experimentally observed in several metamaterials,^[^ ^55^, ^56^, ^57^ ^]^ some sonic crystals^[^ ^58^ ^]^ and photonic crystals,^[^ ^59^, ^60^ ^]^ and also electric circuits.^[^ ^61^, ^62^ ^]^ However, it remains a challenge for the experimental observation of SOTIs in real materials due to the strict requirements of single crystal samples and scanning tunneling microscopy. Up to date, only a few 3D materials have been experimentally evidenced to show SOTI state with 1D hinge states, such as Bi,^[^ ^17^ ^]^ Bi_4_Br_4_,^[^ ^20^ ^]^ and Td‐WTe_2_.^[^ ^63^ ^]^ The detection of corner states has not yet been realized in experiment for 2D materials due to the lack of applicable materials. Fortunately, the high‐quality monolayer 2H‐VS_2_ has already been grown by a molten salt‐mediated precursor system and epitaxial mica growth platform.^[^ ^39^ ^]^ In addition, the semiconductor properties of monolayer 2H‐VS_2_ with a large global band gap have been experimentally confirmed,^[^ ^64^ ^]^ which greatly favor the experimental detection of corner states since these states are well separated from the global bands and the edge states (see Figure 3). These facts show that the spin‐polarized corner states in monolayer 2H‐VS_2_ are promising to be convinced in future experiments.

Quite recently, we come aware of a pioneer work that reported the magnetic SOTI state in two 2D materials including 2H‐RuCl_2_ and Janus VSSe,^[^ ^65^ ^]^ with the occurrence of corner states under different magnetizations. In addition, these materials were found to possess a significant valley polarization effect, nicely bridging the gap between spintronics and valleytronics. Noticing the fact that the monolayer form of 2H‐RuCl_2_ and Janus VSSe has not been realized in experiments makes the experimental observation of these novel properties unavailable. The monolayer 2H‐VS_2_ (and VSe_2_^[^ ^66^ ^]^) proposed in this work has already been synthesized by advanced experiments, and its magnetic ground state has also been confirmed. Thus, the SOTI band structure, the topological corner states, and the potential valley polarization effect proposed in the literature are potentially detected in monolayer 2H‐VS_2_.

## 4 Conclusions

In conclusion, taking the monolayer 2H‐VS_2_ as an example, we theoretically reveal the presence of magnetic SOTI states in a family of transition metal dichalcogenides 2H‐VX_2_ (X = S, Se, Te). The free‐standing monolayer 2H‐VS_2_ is dynamically stable and naturally shows in‐plane FM ordering. There are diverse size band gaps in different spin channels of the 2H‐VX_2_ (X = S, Se, Te), which is a magnetic semiconducting signature. Both band gaps are in topological non‐triviality with corner states possessing quantized fractional charge and ensured by the *C_3_* rotation symmetry. These topological corner states are found to be robust against holes, SOC, and magnetization direction. In addition, the corner states in the 2H‐VX_2_ (X = S, Se, Te) are fully spin‐polarized, which is different from those observed in nonmagnetic SOTIs reported previously. Our findings provide important insights into the high‐order topology in 2D magnetic systems.

## Conflict of Interest

The authors declare no conflict of interest.

## Supporting information

Supporting Information

## Data Availability

Research data is not shared.
