# Supplementary material for: Magnetic Second‐Order Topological Insulators in 2H‐Transition Metal Dichalcogenides
Source: Adv Sci (Weinh). 2023 Jul 30;10(27):2301952. doi: 10.1002/advs.202301952 (PMC10520633; doi:10.1002/advs.202301952)
Supplement: Supplementary file 1 — Supporting Information [file ADVS-10-2301952-s001.pdf]

ADVANCED SCIENCE
Open Access

Supporting Information

for *Adv. Sci.*, DOI 10.1002/advs.202301952

Magnetic Second-Order Topological Insulators in 2H-Transition Metal Dichalcogenides

*Guodong Liu, Haoqian Jiang, Zhenzhou Guo, Xiaoming Zhang, Lei Jin, Cong Liu and Ying Liu**

Supplementary Information for

Magnetic Second-Order Topological Insulators in 2H-Transition Metal Dichalcogenides

I. Ground states and electronic band structure under different U values

Then, the electronic band structure of different U values is shown in Figure S1. We have checked that there are almost no effects on higher-order topological properties of monolayer 2H-VS_2 by changing the U value. The fractional corner charge obtained by the C_3-symmetry eigenvalues is e/3 at different U values. On the other hand, the increase of the U value makes the band gap in the spin-down channel larger (see Table S1). Therefore, the topological properties of monolayer 2H-VS_2 are robust against the effective onsite U values.

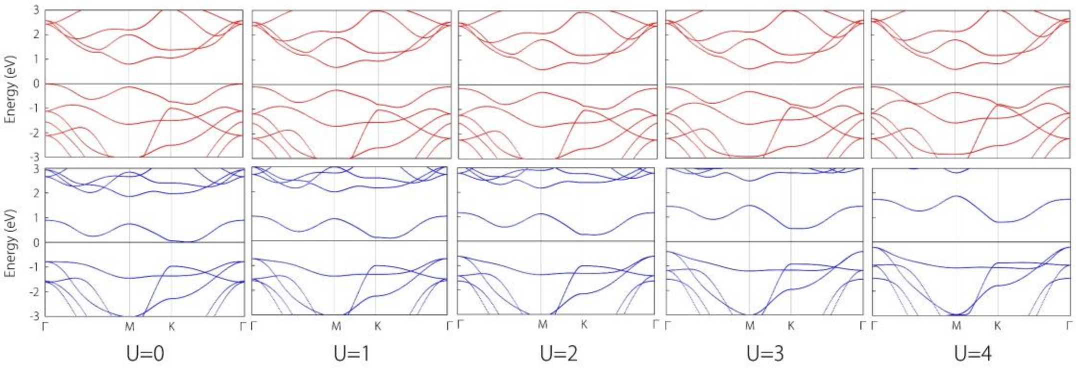

Figure S1. The electron band structures of monolayer 2H-VS_2 at different U values show spin-up on the top panel and spin-down on the bottom panel.

Before studying the electronic band structure, it is necessary to determine the ground states for the monolayer 2H-VS_2. Here, we consider three magnetic configurations, including the ferromagnetic (FM) configuration, and two

antiferromagnetic (AFM) configurations (as shown in Figure S2). Our first-principles calculations show that the lowest energy always occurs for the FM configuration at different U values, as shown in Figure S2.

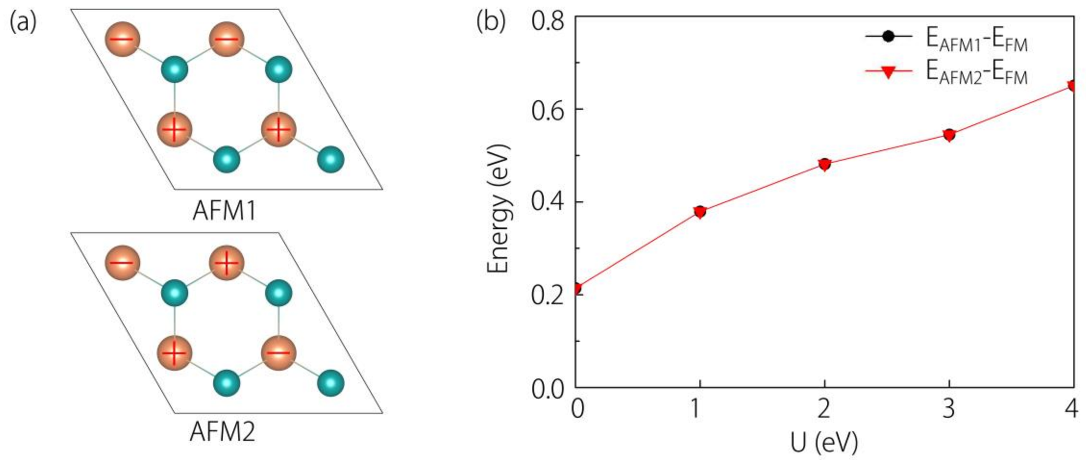

Figure S2. (a) Two antiferromagnetic configurations of the monolayer 2H-VS_2. (b) The energy difference between AFMs and FM state at different U values.

Table S1. Magnetic moment and band gap size of monolayer 2H-VS_2 at different U values.

U	0	1	2	2.5	3	4
V magnetic moment (μ_B)	0.967	1.119	1.246	1.301	1.365	1.509
Total magnetic moment (μ_B)	0.847	0.938	0.992	1.004	1.020	1.060
Gap up (eV)	0.811	0.789	0.765	0.753	0.740	0.713
Gap down (eV)	0.798	0.834	0.885	0.909	0.941	1.021
Gap tot (eV)	0.031	0.210	0.417	0.526	0.646	0.713
Corner charge	$\frac{e}{3}$	$\frac{e}{3}$	$\frac{e}{3}$	$\frac{e}{3}$	$\frac{e}{3}$	$\frac{e}{3}$

II. Magnetic configurations

We plot the 3D magnetic charge density for monolayer 2H-VS_2 in Figure S3. One can derive that the magnetic moments are mainly from the transition metal V atoms. It can be seen that the magnetic ground state of monolayer 2H-VS_2 is FM.

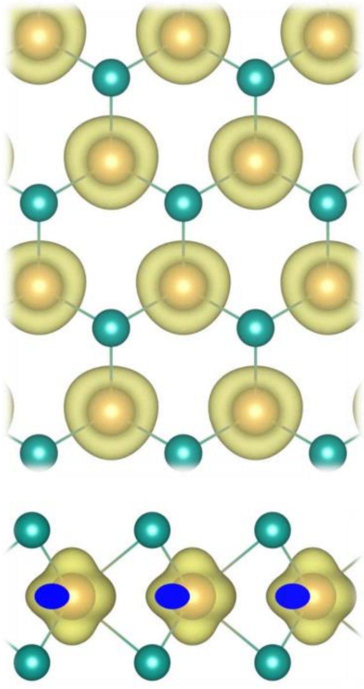

Figure S3. The 3D magnetic charge density of monolayer 2H-VS_2. The top panel is a top view and the bottom panel is a side view. Red and yellow represent V and S atoms, respectively, and the green irregular surface shows the magnetic charge distribution.

III. A non-trivial WC

As a quantized electron center, WC is equivalent to the contribution of electrons to polarization on a macroscopic level. In a trivial insulator, WC coincides with atomic positions because electrons are confined to atomic positions. In non-trivial insulators, the WC position is at the bonding position. In BKL, the non-trivial higher-order topological phase can be further captured by the location of WC. Due to the existence of C_3 symmetry, polarization can be calculated directly from C_3 eigenvalues, thus further obtaining WC. Its position is shown in Figure S4, which is located in the center of the hexagon composed of V and S atoms rather than the atomic position. It can be inferred that monolayer 2H-VS_2 has the characteristics of HOTI.

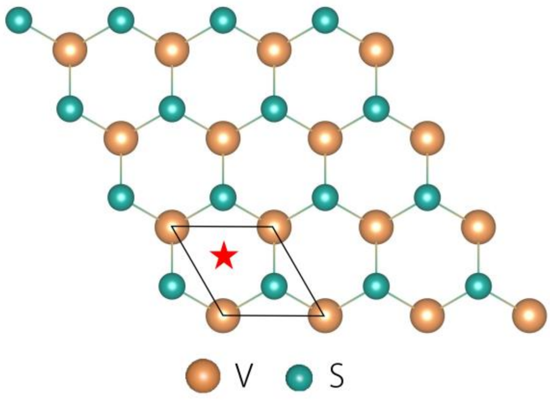

Figure S4. The position of the WC of monolayer 2H-VS_2 is indicated by a pentagram shape.

IV. Electronic band structures and fractional corner charges of other magnetic h-TMD semiconductors

The band structure of other magnetic h-TMD is shown in Figure S5. Furthermore, we performed spin-polarized ab initio molecular dynamics (AIMD) simulations in a large 3×3×1 supercell to assess the thermal stability of monolayer 2H-VSe_2 and monolayer 2H-VTe_2 at room temperature. The results are presented in Figure S6a and Figure S6c, respectively. After 3000 steps at 300 K, we observed no bond breaking or geometric reconstruction in the final state, indicating the thermal stability of 2H-VX_2 (X = S, Se, Te). Additionally, we calculated the phonon spectra of monolayer 2H-VSe_2 and 2H-VTe_2, as shown in Figure S6b and Figure S6d, respectively. Notably, no imaginary frequencies were observed throughout the Brillouin zone, indicating their dynamical stability.

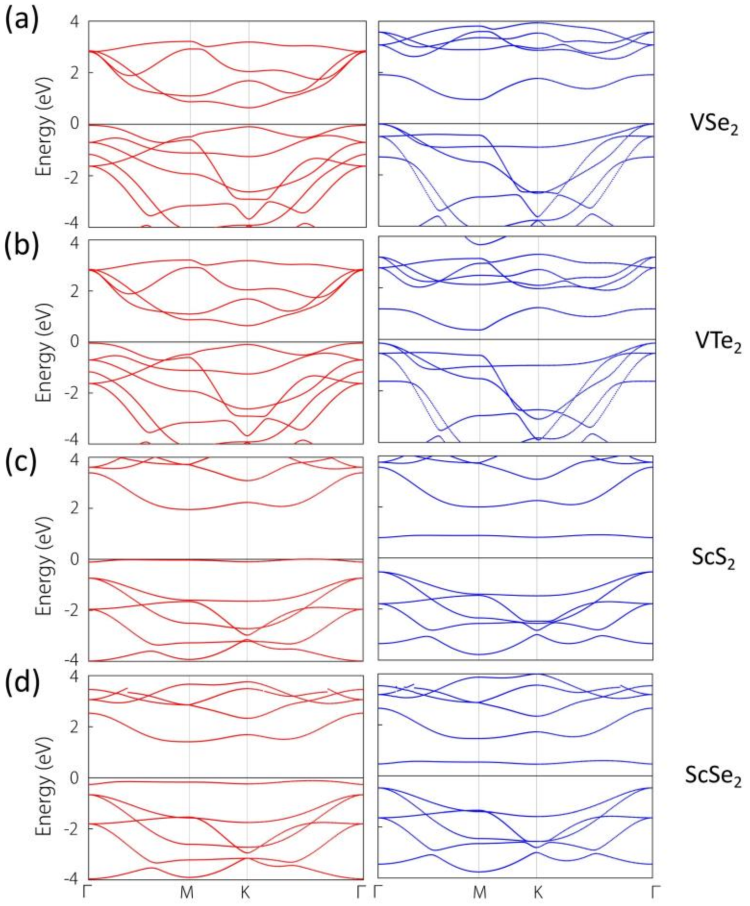

Figure S5. Electron band diagram of magnetic h-TMD material (spin-up on the left

panel, spin-down on the right panel). (a) VSe_2; (b) VTe_2; (c) ScS_2; (d) $ScSe_2$.

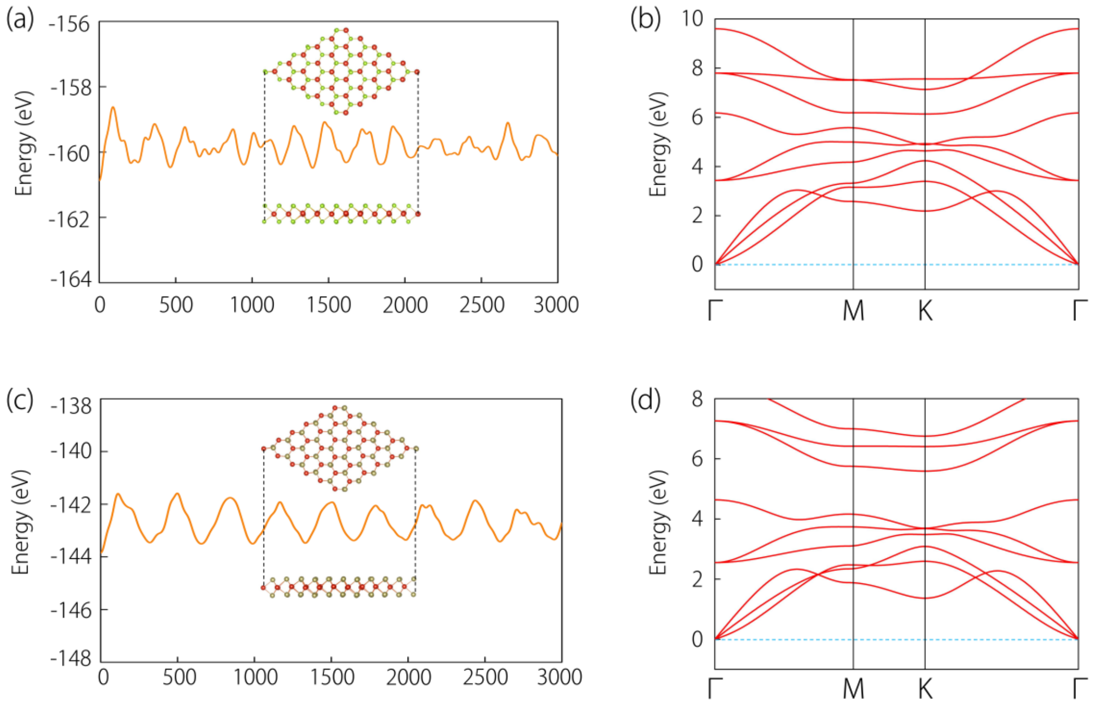

Figure S6. The thermal and dynamical stabilities of the monolayer 2H-VX_2 (X = S, Se, Te). (a) and (c) are the simulated thermal stability of X=Se and X=Te at 300 K AIMD, respectively. (b) and (d) are the phonon spectra calculated for X=Se, Te based on 2×2×1 supercells.

By calculating the C_3-symmetry eigenvalues of these materials, we find that they all have non-zero fractional corner charges, which indicates that they all have higher-order topological properties (see Table S2). The reason why the value of the fractional corner charge in the spin-up of monolayer ScS_2 is 2e/3 is that the value of the fractional corner charge is related to the boundary exposed by the unit cell. To this end, we constructed ScS_2 of two kinds of boundaries, namely, the L_1 type boundary and the L_2 type boundary of the structure in Figure S7 (a) and (c). Due to the difference of C_3 eigenvalues of the two kinds of boundaries, the calculated fractional corner charges are e/3 and 2e/3, respectively.

Table S2. Lattice constants, band gap sizes, Curie temperatures and corner charges of magnetic h-TMD materials.

Material	Lattice Parameter (Å)	Gap up/down (eV)	T_C (K)[36]	$Q^{(3)}_{corner}$ (up/down)
VS_2	a=b=3.173	0.7344/1.2278	292	$\frac{e}{3}/\frac{e}{3}$
VSe_2	a=b=3.343	0.6842/0.9560	472	$\frac{e}{3}/\frac{e}{3}$
VTe_2	a=b=3.598	0.6037/0.5123	553	$\frac{e}{3}/\frac{e}{3}$
ScS_2	a=b=3.788	1.7789/1.3348	--	$\frac{2e}{3}/\frac{e}{3}$
$ScSe_2$	a=b=3.945	1.5243/0.9292	--	$\frac{e}{3}/\frac{e}{3}$

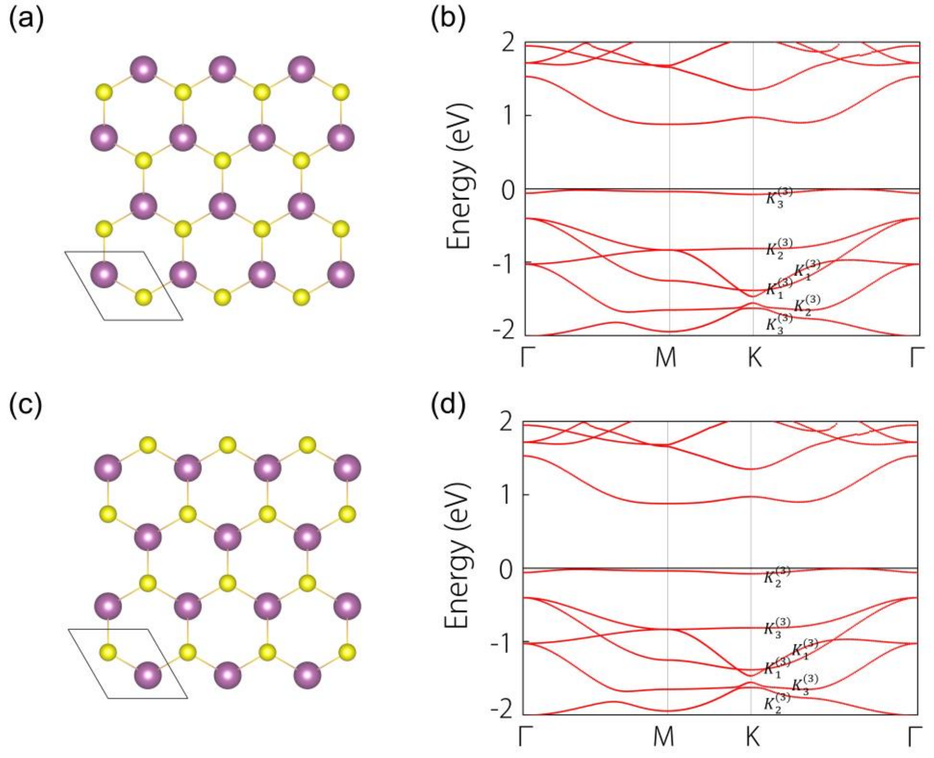

Figure S7. (a), (b) are the lattice structure and electronic band of the L1 boundary, The purple atom is Sc and the yellow atom is S; (c), (d) is the lattice structure and electronic band of the L2 boundary. In the band, we have marked the eigenvalues of the occupation band near the Fermi level.

V. The distribution of corner states in different cases

In order to verify the existence of corner states, we have utilized the tight-binding (TB) method to calculate the energy spectra for both spin-up and spin-down states in triangular nanodisks. To ensure the accuracy of the TB method, we have compared the results obtained from TB calculations with those derived from DFT calculations. Figure S8 shows that the electronic band structures obtained from both methods are in perfect agreement, confirming the validity of the TB method.

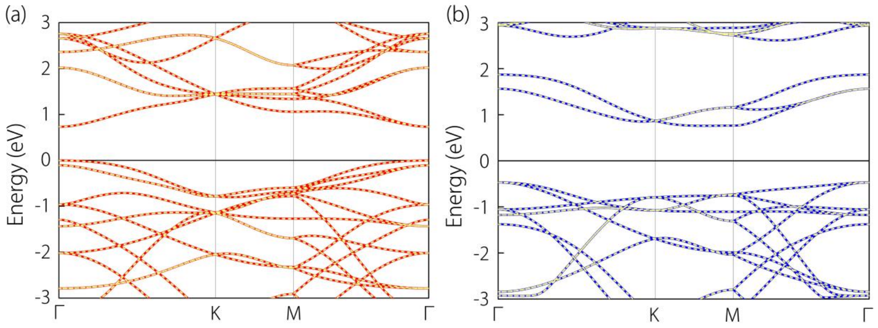

Figure S8. Comparison between the results calculated by TB (dashed lines) and DFT (solid lines) methods for spin-up (a), and spin-down (b) states.

When the boundary of the nanodisk is zigzag, it can be seen from the calculated nonzero polarization that there is still an edge charge at the boundary of one dimension. The presence of edge charge will greatly affect the performance of corner charge, making it impossible to observe meaningful angular state results, as shown in Figure S9. So, in the text, we choose an armchair boundary that polarizes to zero.

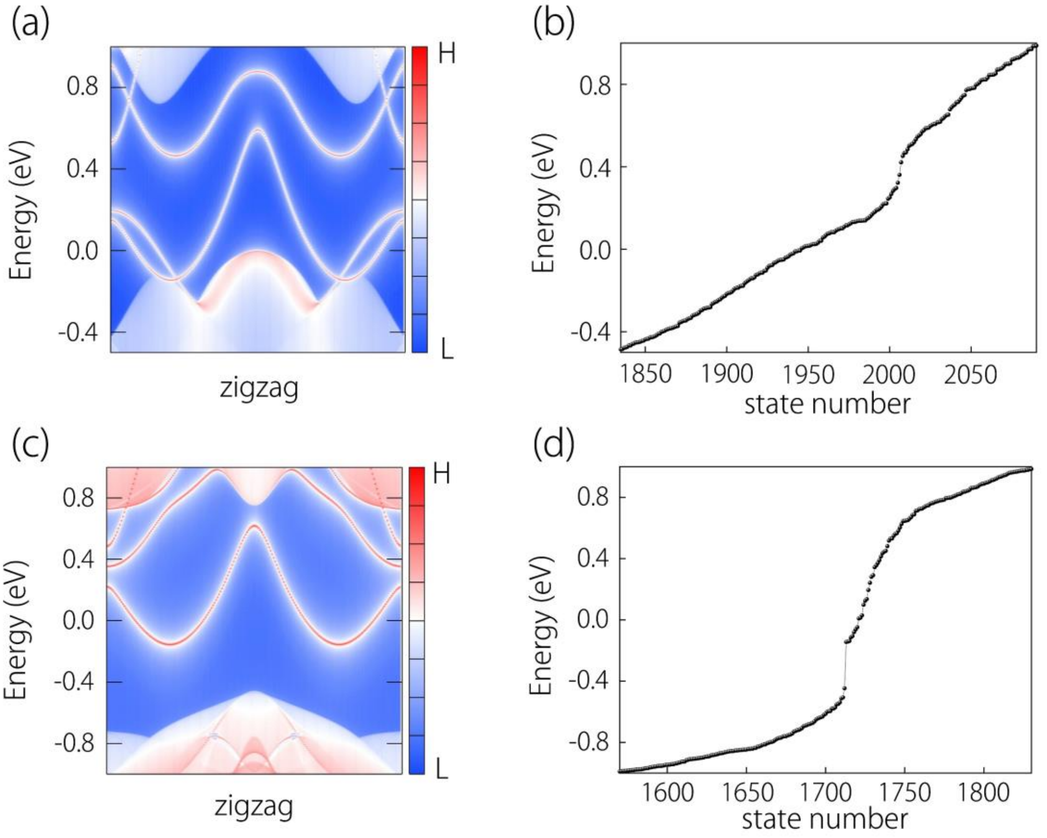

Figure S9. (a), (b) and (c), (d) are the edge states and energy spectra of the zigzag boundary spin-up and spin-down, respectively.

In PDOS without considering SOC, it can be seen that when the spin is up, the highest price to the lowest conduction band is mainly contributed by the d orbital of V; when the spin is down, the highest price band moves up to the conduction band and forms a band gap in the range of 1.8 eV to 2.7 eV, and its edge state is shown in Figure S10a. Therefore, we conclude that this band gap should have the same properties as the spin-up gap. The results shown in Figure S10b also verify this inference and prove the correctness of our calculation results.

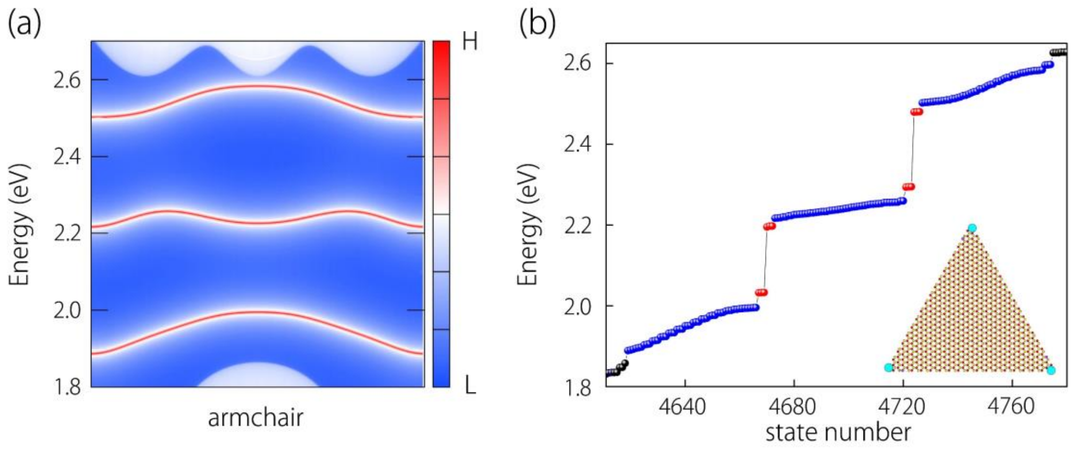

Figure S10. (a) The edge state of spin-down is in the range of 1.8 eV to 2.7 eV when the boundary is armchair type. (b) is the spectrum corresponding to (a) and the distribution of corner states in real space.

Because the size of the nanodisk will affect the corner state, it is necessary to confirm that the corner state has excluded the finite size effect when observing the corner state. For concreteness, if the sample is too small, the corner states may couple with each other, with their wave functions less localized and their energies pushed away from the band gap. In addition, the size of the sample will exert an effect on the energy splitting of the corner states, in general, with the increasing size one may notice that the energy splitting for the corner states becomes smaller and smaller. By expanding the nanodisk and calculating the distribution of the corner state again, we found that the degeneracy and energy of the corner state did not change, as shown in Figure S11. Therefore, we can judge that the size of the nanodisk we selected has excluded the effective size effect.

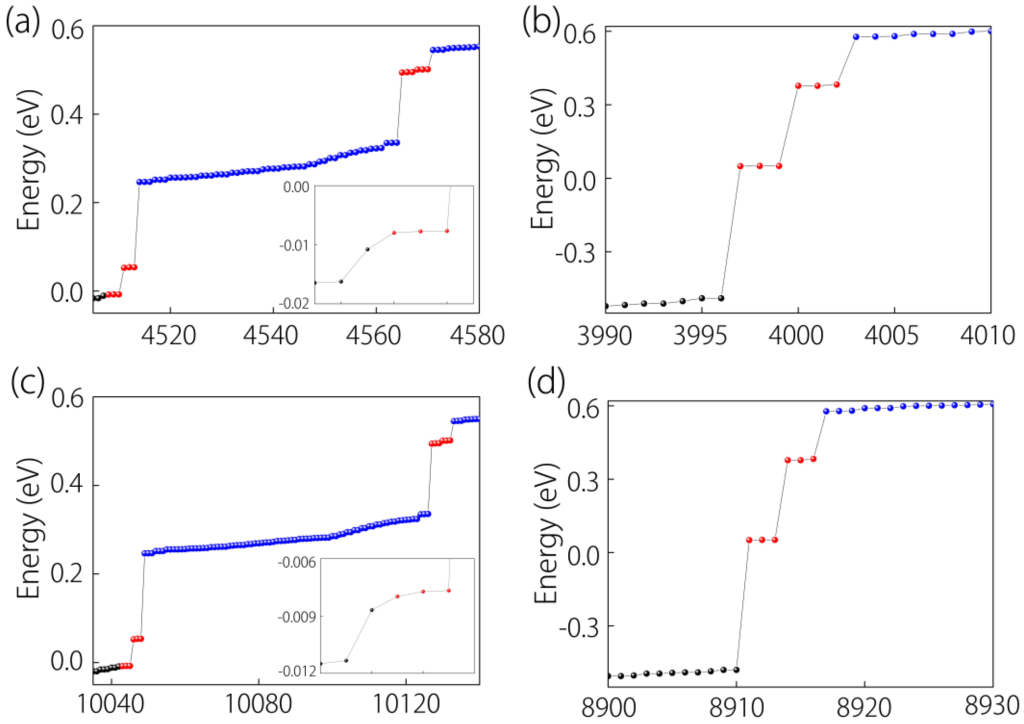

Figure S11. (a) (b) and (c) (d) are the spin-up and spin-down corner state distributions at the size of 10 nm and 15 nm, respectively.

The distribution of electronic states after the introduction of defects in the triangular nanodisk is shown in Figure S12. As a result of the exposed dangling bond in the defect space, electrons can be observed to accumulate at the defect. But this does not affect the distribution of corner states, which also proves the robustness of corner states.

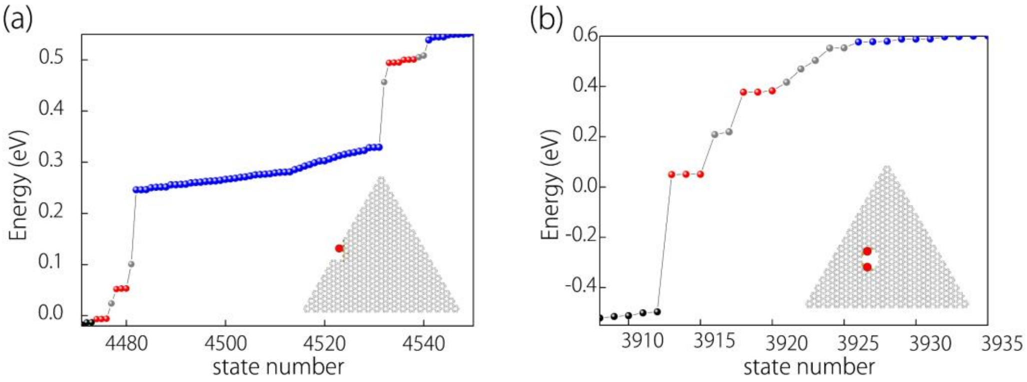

Figure S12. (a) and (b) are spin-up and spin-down energy spectra after the introduction of defects, respectively. The green dots show the distribution of electrons caused by the exposed dangling bond at the defect.

VI. Influence of magnetization direction on the band gap

In the analysis of the topological origin of corner states, various crystal symmetries are commonly employed. However, these symmetries do not need to be precisely preserved. This is because corner states, as topological domain wall modes between edges, must exhibit robustness against perturbations. In other words, once corner states exist, they cannot be completely annihilated without a substantial energy cost. This is a fundamental characteristic of second-order topological insulators (SOTIs).

It is widely recognized that closing and reopening the band gap or breaking symmetries are two ways to change the topological classification of material. If two quantum phases can transform into each other without closing the band gap, they are considered topologically equivalent. To verify the topological equivalence when the magnetization direction changes from out-of-plane to in-plane and the C_3 symmetry of the system is broken, we calculated the variation in the band gap as a function of magnetization direction, as shown in Figure S13. It can be observed that the band gap gradually decreases, but the range of change is relatively small, approximately 2 meV. Based on this observation, we can predict that the topology remains equivalent in both cases, and the system with in-plane magnetization still exhibits SOTI behavior.

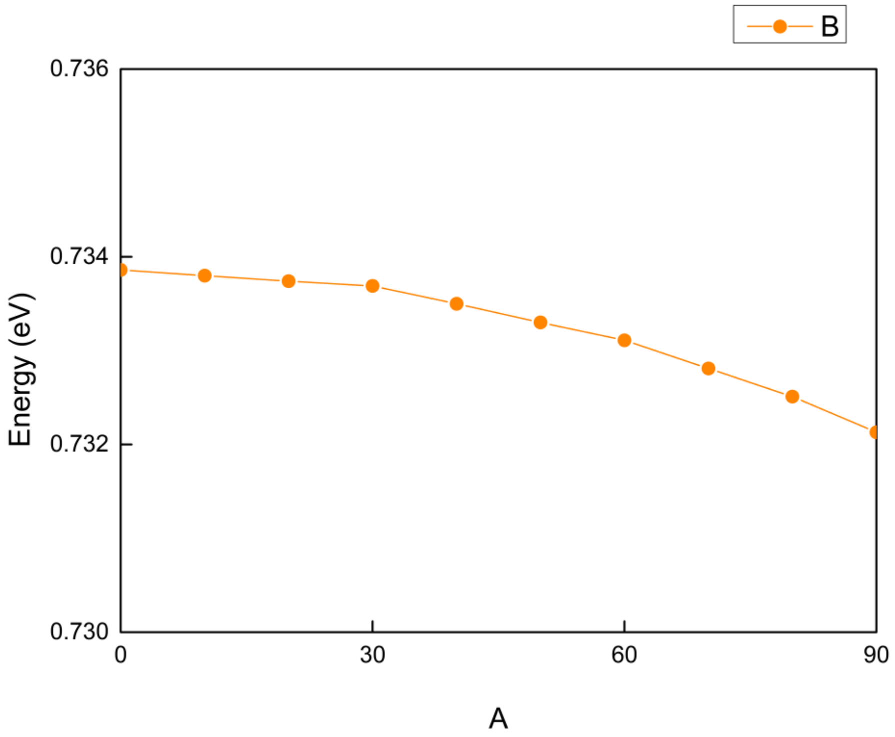

Figure S13. The change of band gap when the direction of magnetization changes from out-of-plane to in-plane.
